# Investigating the potential of triclinic ABSe_3_ (A = Li, Na, K, Rb, Cs; B = Si, Ge, Sn) perovskites as a new class of lead-free photovoltaic materials

**DOI:** 10.1038/s41598-024-72555-0

**Published:** 2024-09-30

**Authors:** Eman Khalafalla Mahmoud, S. I. El-dek, Ahmed A. Farghali, Mohamed Taha

**Affiliations:** Materials Science and Nanotechnology Department, Faculty of Postgraduate Studies for Advanced Sciences (PSAS), Beni-Suef University (BSU), Beni-Suef, Egypt

**Keywords:** Chalcogenide perovskites, Density functional theory, Electronic properties, Optical properties, Solar cell applications, Chemistry, Optics and photonics, Materials science, Condensed-matter physics, Materials for devices, Materials for energy and catalysis, Materials for optics, Nanoscale materials, Structural materials, Theory and computation

## Abstract

In recent years, chalcogenide perovskites have emerged as promising candidates with favorable structural, electrical, and optical properties for photovoltaic applications. This paper explores the structural, electronic, and optical characteristics of ABSe_3_ perovskites (where A = Li, Na, K, Rb, Cs; B = Si, Ge, Sn) in their triclinic crystallographic phases using density functional theory. The stability of these materials is ensured by calculating formation energies, tolerance factors (*T*_*f*_), and phonon dispersion. The *E*_form_ values of all ABSe_3_ are negative, suggesting favorable thermodynamic stability. The *T*_*f*_ values range between 0.82 and 1.1, which is consistent with stable perovskites. The phonon dispersion analysis of the chalcogenide perovskites revealed no imaginary frequencies in any of the vibrational modes, confirming their stability. The electronic band structures and corresponding density of states are computed to unveil the semiconducting nature of the studied compounds. These perovskites are promising for high-performance solar cells due to their indirect bandgaps (*E*_g_, 1.10–2.33 eV) and a small difference between these indirect and direct gaps (0.149–0.493 eV). The *E*_g_ values increase as the ionic radii of A-site elements increase (Li < Na < K < Rb < Cs). At the B-site, Si-based chalcogenides have the largest *E*_g_ values, followed by Sn-based and then Ge-based materials. Furthermore, optical properties such as the real part and imaginary part of the dielectric function, refractive index extinction coefficient, optical conductivity, absorption coefficient, reflectivity, and energy loss are predicted within the energy range of 0–50 eV. Several ABSe_3_ materials, particularly LiGeSe_3_ and NaGeSe_3_, demonstrated optical properties comparable to both traditional and emerging materials, suggesting their potential for effective use in solar cells.

## Introduction

Due to the increasing global energy use, and as currently available energy sources are mostly based on fossil fuels, researchers are increasingly focused on developing renewable energy resources^1,2^. Solar energy, a vital source of renewable energy, can be harnessed through thermoelectric and photovoltaic technologies. These technologies convert heat and light from the sun into electrical energy^3^. The selection of materials is crucial for determining their suitability in thermal applications (e.g., thermoelectric devices) and photoelectric applications (e.g., solar cells) due to their property dependence (conductivity, band gap)^4^.

Perovskite materials may play a crucial role in this regard because of their extraordinary physical properties such as magnetic, ferroelectric, optical, charge ordering, spin-dependent transport and high thermoelectric power^5,6^. Within the diverse perovskite family (oxide, halide, and chalcogenide)^7–13^, chalcogenide perovskites, with a general formula ABX_3_ (where A and B represent cations of different sizes and X is a chalcogenide element S, Se, or Te), are attracting attention for their potential in solar energy conversion due to their favorable structural, optical, and electronic characteristics. Oxide and halide perovskites have long been exhibiting remarkable properties for solar energy conversion^14–16^. For instance, various perovskite materials displayed promise in several technological applications. Compounds like A_2_NiMnO_6_ (A = La, Gd)^17^, Ca_2_NaIO_6_^18^, Sr_2_NaIO_6_^18^, RbPbBr_3_^19^, RbPbI_3_^19^, and Rb_2_NaCoX_6_ (X = Cl, Br, I)^20^ exhibit properties suitable for magneto-caloric refrigeration, photocatalysis, thermoelectric energy conversion, and other applications due to their favorable structural, electrical, optical, and transport characteristics. However, oxide perovskites often exhibit limitations for solar energy conversion due to their wide band gaps^21^. This limitation arises from the high electronegativity of oxygen, which typically results in bandgaps positioned in the ultraviolet (UV) region. On the other hand, halide perovskites suffer from toxicity, moisture, and thermal instability^22^.

Lead-based perovskites have attracted considerable interest for their outstanding optoelectronic properties, particularly in solar cell applications. However, the toxicity of lead raises significant environmental and health concerns, prompting researchers to explore lead-free alternatives^5,11^. The development of environmentally friendly lead-free perovskites solar cells has become crucial to realize their large-scale production. To address this challenge, several novel lead-free perovskite materials have been explored both computationally and experimentally^23–26^. A computational study using density functional theory (DFT) of ethylammonium tin chloride (C_2_H_5_NH_3_SnCl_3_) has shown that this material can exhibit favorable electronic band structure and optical properties, making it suitable for thermoelectric applications^27^. Recent computational studies, such as that by Riku et al.^28^, have identified lead-free ABX_3_ (A = Rb or Cs, B = Sn, Sr, or Ca, X = Cl, Br, or I) perovskites are ideal for solar cells and light-emitting diodes^28^. Sarker et al. demonstrate that NaGeX_3_ (X = F, Cl, Br, and I) is beneficial and non-toxic for solar cell applications^29^. DFT computation of KSnI_3_, KSn_0.5_Ge_0.5_I_3_, and KGeI_3_ demonstrate their capability as a functional layer in nontoxic perovskite solar cells^30^.

Chalcogenide perovskites offer superior environmental stability compared to halide perovskites, making them ideal for long-term applications like solar cells, where exposure to moisture and heat can degrade performance^31^. They are also composed of non-toxic elements, making them more environmentally friendly and safer for commercial use^1,7,31^. Chalcogenide perovskites can achieve high absorption coefficients, which enhances their efficiency as photoabsorbers^32^. Their covalent nature improves electronic and optical performance, leading to better charge transport and reduced recombination losses^33^. Additionally, they possess higher thermal and moisture stability than oxide perovskites, crucial for maintaining device performance^31^. Finally, chalcogenide perovskites offer tunable bandgap structures, optimizing their performance for applications like tandem solar cells^23,33^.

Recently, a diverse range of chalcogenide perovskites with interesting properties has been both experimentally verified and theoretically predicted^31,34–39^. Perera et al.^40^ reported the successful synthesis of several chalcogenide perovskites, including BaZrS_3_, CaZrS_3_, SrZrS_3_, and SrTiS_3_. These materials exhibit unique physical properties, such as being free of deep-level defects, which is beneficial for energy harvesting and other optoelectronic applications. Shaili et al. reported that synthesized CaSnS_3_ exhibits a suitable bandgap of 1.72 eV for optoelectronic applications^41^. SrZrS_3_ and SrZrSe_3_ may be employed as a photovoltaic absorber coating inside solar cells, as well as in energy conversion devices owing to their acceptable optical and thermoelectric properties^42^. Naincy et al. predicted the applicability of BaZrS_3_ and BaZrSe_3_ in energy conversion device fabrication^43^. Nishigaki et al. reported the synthesis of BaZrS_3_, SrZrS_3_, BaHfS_3_, and SrHfS_3_, distorted chalcogenide perovskites, with a direct bandgap (1.94–2.41 eV), making them suitable solar cell light absorbers^44^. Furthermore, Majhi synthesized FeNiSe_2_ and FeNiSSe as efficient electrocatalysts for water splitting^45^.

DFT studies have yielded promising chalcogenide perovskite candidates for solar cells. Sun et al.^46^ identified CaTiS_3_, BaZrS_3_, CaZrSe_3_, and CaHfSe_3_ as promising candidates for single-junction solar cells. Similarly, Du et al.^47,48^ proposed Ca_3_Sn_2_S_3_ and ACeTe_3_ (A = Ca, Sr, Ba) as suitable photovoltaic materials. Likewise, Thakur et al.^49^ and Liu et al.^50^ predicted the potential of ABX_3_ (A = Ba, B = Zr, X = S and/or Se) and ABSe_3_ (A = Ca, Sr, Ba; B = Hf, Zr) for photovoltaic applications, respectively. Zhang et al.^51^ further extended this investigation to LaScSe_3_ for optoelectronic applications. Earlier research has highlighted the potential of chalcogenide perovskites, ABX_3_ (A = Ca, Sr, or Ba; B = Ti, Zr, or Hf; and X = O, S, or Se) to address the limitations of halide perovskites, particularly regarding concerns about toxicity and stability^52^.

In this paper, we employ DFT to predict the structural, electronic, and optical properties of chalcogenide perovskites ABSe_3_ (A = Li, Na, K, Rb, Cs; and B = Si, Ge, Sn) in triclinic crystallographic phases. The motivation for this study stems from the growing interest in chalcogenide perovskites due to their potential applications in optoelectronics and photovoltaics. Due to the absence of experimental data for comparison with theoretical results on the investigated perovskites, we employed the Heyd-Scuseria-Ernzerhof hybrid functional (HSE06)^53^. It is a hybrid density functional that combines the strengths of Hartree–Fock theory with DFT. It is particularly designed to address some of the limitations associated with local density approximations (LDA) and generalized gradient approximations (GGA), which often underestimate band gaps in semiconductors and insulators. HSE06 is a powerful tool in computational materials science, providing improved accuracy for electronic structure calculations while also presenting some limitations related to computational cost. HSE06 has been shown to accurately predict bandgaps, electrical, and optical properties for a variety of perovskite materials^54^.

## Computational method

All calculations were carried out with the CASTEP code^55^ within the Materials Studio 2020 package, employing DFT. CsSnS_3_ (triclinic, P-1,2) was taken from the material project website (mp-561710)^56^. Cs atom was systematically replaced with Li, Na, K, and Rb, while Sn atom was substituted with Si and Ge atoms, and the S atoms were replaced with Se atoms. Geometry optimization, electronic, and optical properties, were conducted using HSE06 hybrid functional^53^. Each exchange–correlation (XC) functional has been calculated using kinetic energy cut-offs of 650 eV with norm-conserving pseudopotential^57^. The self-consistent field (SCF) tolerance was 1.0 × 10^−6^ (eV/atom). Furthermore, the total energy tolerance, maximum ionic Hellmann–Feynman force, and maximum ionic displacement tolerance were 1.0 × 10^−5^ (eV/atom), 0.03 (eV/Å) and 0.001 (Å), respectively. A dense $12\times 12\times 12$ Monkhorst and Pack mesh grid was used for k-points sampling of the first Brillouin zone. The phonon dispersion curves for the chalcogenide perovskites were calculated using the GGA/PBEsol method^58^.

The optical properties were calculated using HSE functional by the complex dielectric function $\varepsilon (\omega )$ given as^59^,

1 $$\varepsilon \left(\omega \right)={\varepsilon }_{1}\left(\omega \right)+i{\varepsilon }_{2}\left(\omega \right),$$

where ${\varepsilon }_{1}(\omega )$ and ${\varepsilon }_{2}(\omega )$ represents the real and imaginary part of the dielectric constant. The well-known Kramer–Kronig relation connects the real and imaginary sections of the dielectric functions as follows^60,61^:

2 $$\varepsilon_{1} \left( \omega \right) = 1 + \frac{2}{\pi }P\mathop \int \limits_{0}^{\infty } \frac{{\omega^{\prime} \varepsilon_{2} \left( \omega \right)}}{{\omega^{{\prime}2} - \omega^{2} }}{\text{d}}\omega ,$$

where *P* denotes the principal value of the integral.

3 $${\varepsilon }_{2}\left(\omega \right)=\frac{2{e}^{2}\pi }{\Omega {\varepsilon }_{0}}\sum_{k,v,c}\left|{\psi }_{k}^{c}\right|u.r{\left|{\psi }_{k}^{\upsilon }\right|}^{2}\updelta \left({E}_{k}^{c}+{E}_{k}^{\upsilon }-\text{E}\right).$$

Here, u and e represent the polarization of the incident electric field and the electric charge, respectively. Ω denotes the volume of a unit cell. ${\psi }_{k}^{c}$ and ${\psi }_{k}^{\upsilon }$ signify the wave functions of the valence band and conduction band, respectively, at the position *k*. By using ${\varepsilon }_{1}\left(\omega \right)$ and ${\varepsilon }_{2}(\omega )$, optical properties like reflectivity *R*(ω), absorption coefficient α(ω), optical conductivity σ(ω), refractive index *n*(ω), extinction coefficient *k*(ω) and energy-loss spectrum *L*(ω) are evaluated as follows^62–64^:

4 $$R\left(\omega \right)={\left|\frac{\sqrt{\varepsilon \left(\omega \right)}-1}{\sqrt{\varepsilon \left(\omega \right)}+1}\right|}^{2},$$

5 $$\alpha \left(\omega \right)=\frac{\omega }{c}\sqrt{2\left(\sqrt{{\varepsilon }_{1}^{2}\left(\omega \right)+{\varepsilon }_{2}^{2}\left(\omega \right)}-{\varepsilon }_{1}\left(\omega \right)\right)},$$

6 $$\sigma \left(\omega \right)=\frac{i\omega }{4\pi }\varepsilon \left(\omega \right),$$

7 $$n\left(\omega \right)=\sqrt{2\frac{\sqrt{{\varepsilon }_{1}^{2}\left(\omega \right)+{\varepsilon }_{2}^{2}\left(\omega \right)}+{\varepsilon }_{1}\left(\omega \right)}{2}},$$

8 $$k\left(\omega \right)=\sqrt{2\frac{\sqrt{{\varepsilon }_{1}^{2}\left(\omega \right)+{\varepsilon }_{2}^{2}\left(\omega \right)}-{\varepsilon }_{1}\left(\omega \right)}{2}} ,$$

9 $$L\left(\omega \right)=\frac{{\varepsilon }_{2}\left(\omega \right)}{{\varepsilon }_{1}\left(\omega \right)+{\varepsilon }_{2}\left(\omega \right)}.$$

## Results and discussion

### Structural properties

**Fig. 1:**
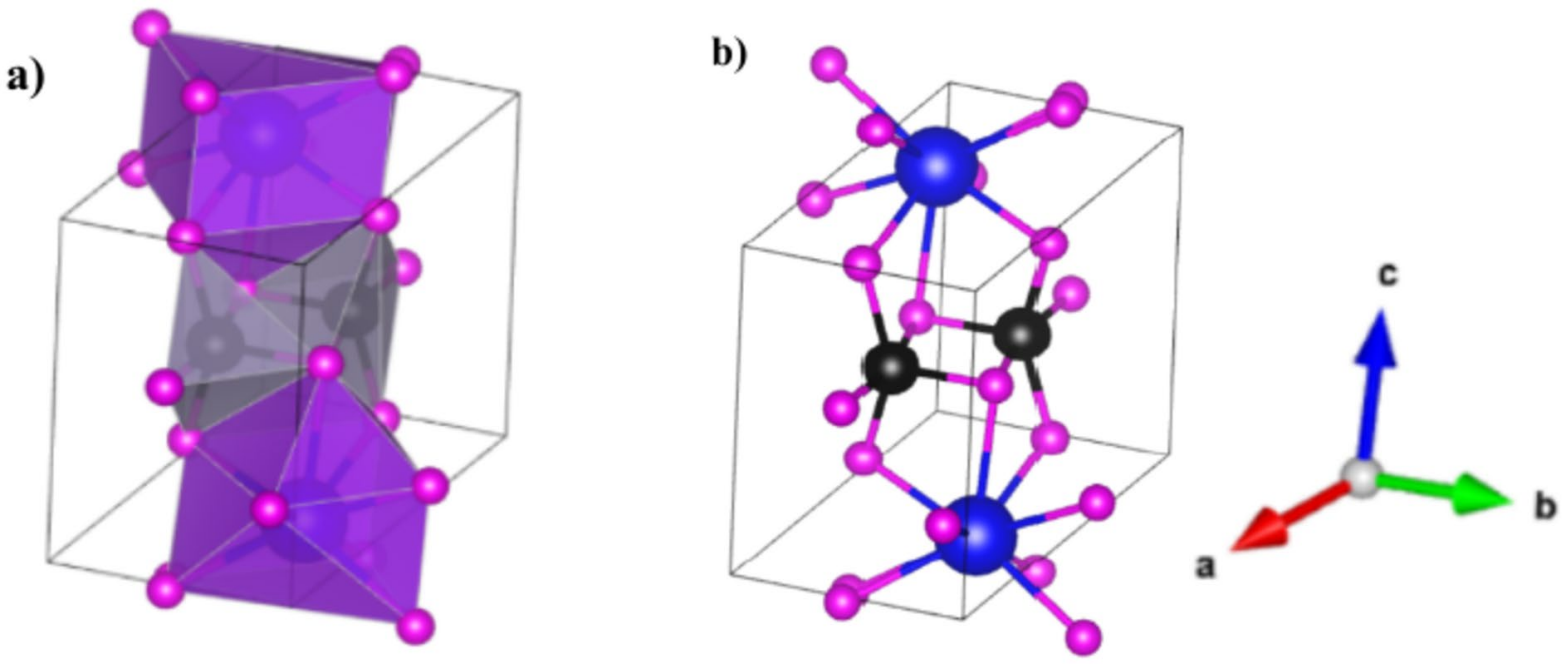
Polyhedral model (**a**) and ball and stick model (**b**) of ABSe_3_. Blue, black and purple are associated with A, B, and Se atoms.

Figure 1 represents the triclinic crystal structure of the chalcogenide perovskite series ABSe_3_ (A = Li, Na, K, Rb, Cs; B = Si, Ge, Sn). The A-site cation exhibits a larger ionic radius compared to the B-site cation, consistent with the typical structural features of perovskites. For systematic analysis, the investigated chalcogenide perovskites were categorized into three groups based on the B-site cation: Si-based (ASiSe_3_), Ge-based (AGeSe_3_), and Sn-based (ASnSe_3_).

The substitutions at the A, B and X sites can cause significant improvements in stability and performance^65,66^. However, by substitutions, the crystal structure of a perovskite material can alter significantly, which can be anticipated using the Goldschmidt tolerance factor (*T*_*f*_)^67^. The *T*_*f*_, Eq. (8), anticipates the viability of a combination of A, B, and X elements to form a perovskite phase by calculating a structural ratio; a value between 0.80 and 1.11 indicates ideal cubic structures or a distorted perovskite structure with tilted octahedral. Structures having *T*_*f*_ values outside the range cannot be considered stable perovskites^68–70^.

10 $${T}_{f}=\frac{{R}_{A}+{R}_{Se}}{\sqrt{2}{ (R}_{B}+{R}_{Se})}.$$

*R*_*A*_, *R*_*B*_, and *R*_*Se*_ symbolize the Shannon ionic radii of A, B, and Se elements, respectively^71^. Our investigated perovskites have tolerance factor values ranging from 0.8158 to 1.117 (Table 1), revealing structural stability.

**Table 1:** Goldschmidt’s tolerance factor (*T*_*f*_) and formation energy (*E*_*form*_) for Si-based, Ge-based, and Sn-based chalcogenides using the HSE06 method.

Perovskite	*T*_*f*_	*E*_*form*_	Perovskite	*T*_*f*_	*E*_*form*_	Perovskite	*T*_*f*_	*E*_*form*_
LiSiSe_3_	0.9153	− 3.84	LiGeSe_3_	0.8700	− 3.30	LiSnSe_3_	0.8158	− 4.17
NaSiSe_3_	0.9717	− 4.54	NaGeSe_3_	0.9213	− 3.99	NaSnSe_3_	0.8662	− 4.70
KSiSe_3_	1.0490	− 5.10	KGeSe_3_	0.9946	− 4.49	KSnSe_3_	0.8874	− 5.17
RbSiSe_3_	1.0728	− 5.11	RbGeSe_3_	1.0226	− 4.56	RbSnSe_3_	0.9562	− 5.29
CsSiSe_3_	1.117	− 5.27	CsGeSe_3_	1.0621	− 4.70	CsSnSe_3_	0.9960	− 5.49

Formation energy (*E*_*form*_), which represents the energy required to form a material from its constituent elements, was utilized to assess the thermodynamic stability of the perovskites by Eq. (11)^72^,

11 $${E}_{form}={E}_{{\text{ABSe}}_{3}}-{\left({E}_{\text{A}}+{E}_{\text{B}}+{3E}_{\text{Se}}\right)}_{\text{atom}}.$$

The energies of A, B, and Se atoms in their corresponding bulk crystal phases are denoted by E_A_, E_B_ and E_Se_, respectively. ${E}_{{ABSe}_{3}}$ is the energy of the ABSe_3_ unit cell. Materials having low (negative) formation energies are more stable and less prone to break down into their constituent elements. This stability is essential for sustaining the efficiency of perovskite solar cells over time, particularly in conditions of operation^73^. This stability is critical for assuring the longevity and reliability of perovskite solar cells, which are frequently subjected to changing environmental conditions. The formation energy also influences the development of tandem solar cells, which combine perovskites with silicon or other materials. Perovskite/silicon tandem cells, for example, have achieved efficiencies of more than 31%, demonstrating the potential for optimizing formation energies in multi-junction configurations^74^. For all studied ABSe_3_, *E*_*form*_ was calculated to be negative (Table 1), suggesting favorable thermodynamic stability. Interestingly, within each series (Si, Ge, or Sn), the formation energies exhibit a trend of becoming more negative (increasingly stable) as the A-site cation follows (Li < Cs < Rb < K < Na). Within each A-site cation, the formation energy tends to become more negative as the B-site cation changes from Sn to Ge to Si.

Lattice parameters, unit cell volume (V), and theoretical density (D) were calculated and presented in Table 2. The increasing trend in unit cell volume coincides with an increase in lattice parameters, as a result of the increase in the ionic radius of the A-site cation.

**Table 2:** The Lattice parameters, unit cell volume, and theoretical density (D) of the studied perovskites using the HSE06 method.

Perovskite	Lattice parameters						V (Å^3^)	D (g/cm^3^)
	A (Å)	B (Å)	c(Å)	α°	β°	γ°		
LiSiSe_3_	5.926	5.264	7.838	101.226	112.367	113.288	189.829	4.757
NaSiSe_3_	6.544	5.600	7.323	93.536	114.181	119.514	201.029	4.757
KSiSe_3_	6.248	5.918	7.294	9.9151	15.799	8.940	226.625	4.455
RbSiSe_3_	6.334	6.191	7.224	89.567	115.697	107.709	240.692	4.835
CsSiSe_3_	6.706	7.182	7.586	97.726	114.212	106.905	305.078	4.331
LiGeSe_3_	6.061	5.306	8.060	100.741	113.470	14.350	196.654	5.343
NaGeSe_3_	6.627	5.634	7.449	93.271	114.833	118.518	209.912	5.259
KGeSe_3_	6.405	6.196	7.157	92.532	116.33	105.501	240.936	4.804
RbGeSe_3_	6.517	6.127	7.412	89.737	115.839	108.933	248.726	5.273
CsGeSe_3_	6.603	6.536	7.245	89.156	116.598	104.494	269.014	5.461
LiSnSe_3_	6.240	5.192	8.241	102.604	112.814	112.844	204.344	5.891
NaSnSe_3_	6.352	5.410	8.144	96.431	114.887	115.322	214.496	5.861
KSnSe_3_	6.434	5.848	7.986	89.083	116.404	110.644	248.337	5.278
RbSnSe_3_	6.664	6.550	7.472	97.489	114.526	106.341	273.139	5.362
CsSnSe_3_	6.733	6.437	7.792	88.384	116.098	109.081	283.970	5.712

### Phonon dispersion

**Fig. 2:**
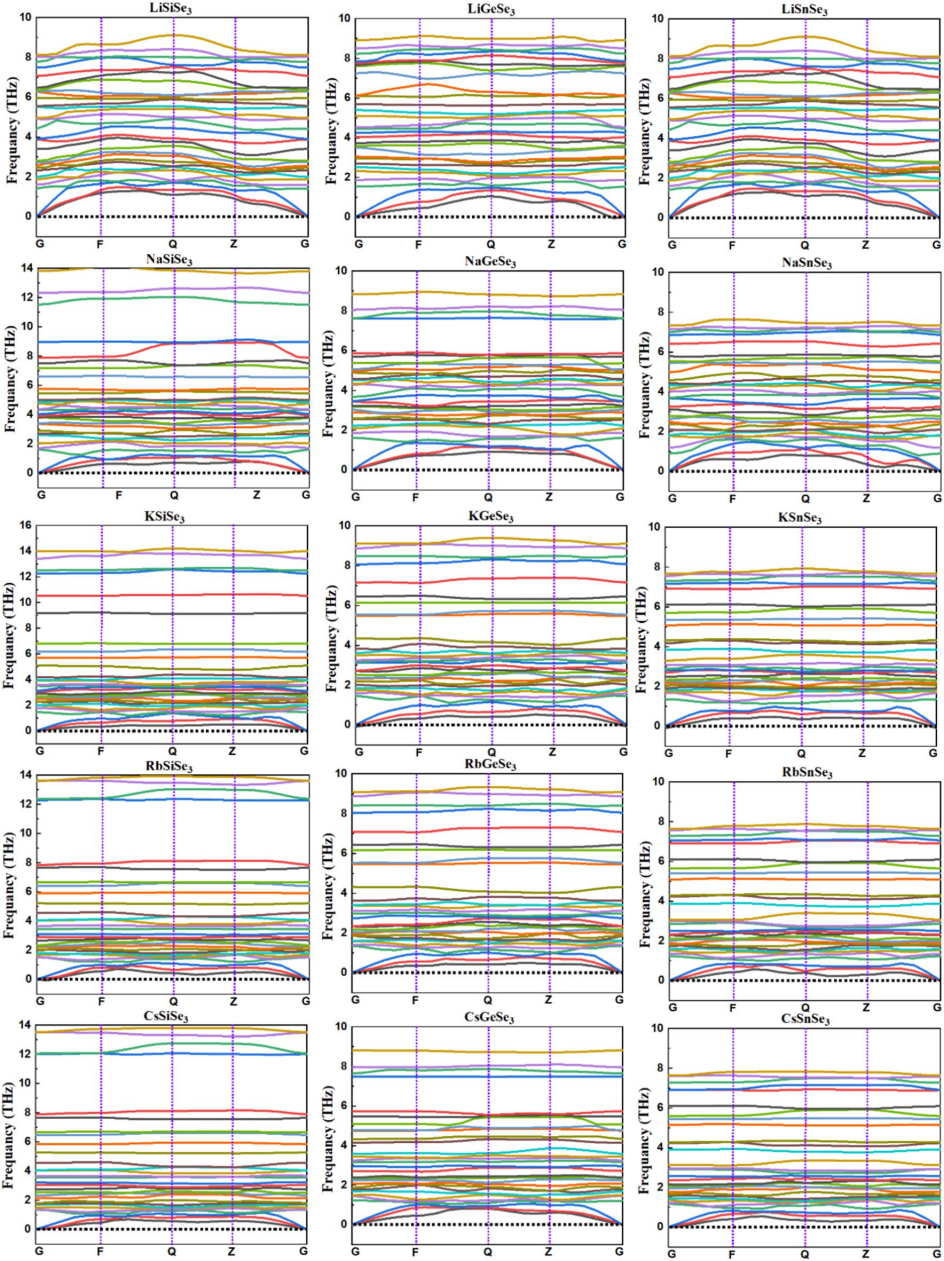
Phonon dispersion curves for the chalcogenide perovskites ABSe_3_ by using the GGA/PBEsol method.

The calculation of phonon dispersion curves provides valuable insights into the dynamical stability of materials by revealing the vibrational frequencies of atoms within the crystal lattice. The phonon dispersion of a material significantly influences its mechanical properties, such as elasticity and hardness, as well as its thermal behavior, including thermal conductivity and specific heat capacity. These factors collectively determine its overall suitability for practical applications, such as structural components and electronic devices^75,76^. The presence of imaginary frequencies in phonon dispersion curves indicates dynamic instability of the material, whereas their absence signifies stability. Figure 2 depicts the calculated phonon dispersion curves for the chalcogenide perovskites, revealing the absence of imaginary frequencies across all vibrational modes, indicative of their stability^77^. One remarkable feature from Fig. 2 is the band gap in the phonon spectrum. We can see that this band gap was noticed for NaSiSe_3_, NaGeSe_3_, KSiSe_3_, KGeSe_3_, RbSiSe_3_, RbGeSe_3_, CsSiSe_3_ and CsGeSe_3_ meanwhile extremely small for other perovskites. The presence of phonon band gaps, regions in the dispersion where no phonon modes exist, can lead to a reduction in thermal conductivity due to the limited number of available heat-carrying channels^77^. Therefore, we can conclude that the above-mentioned perovskites have lower thermal conductivity compared to the other compounds.

### Electronic properties

#### Band structures

**Fig. 3:**
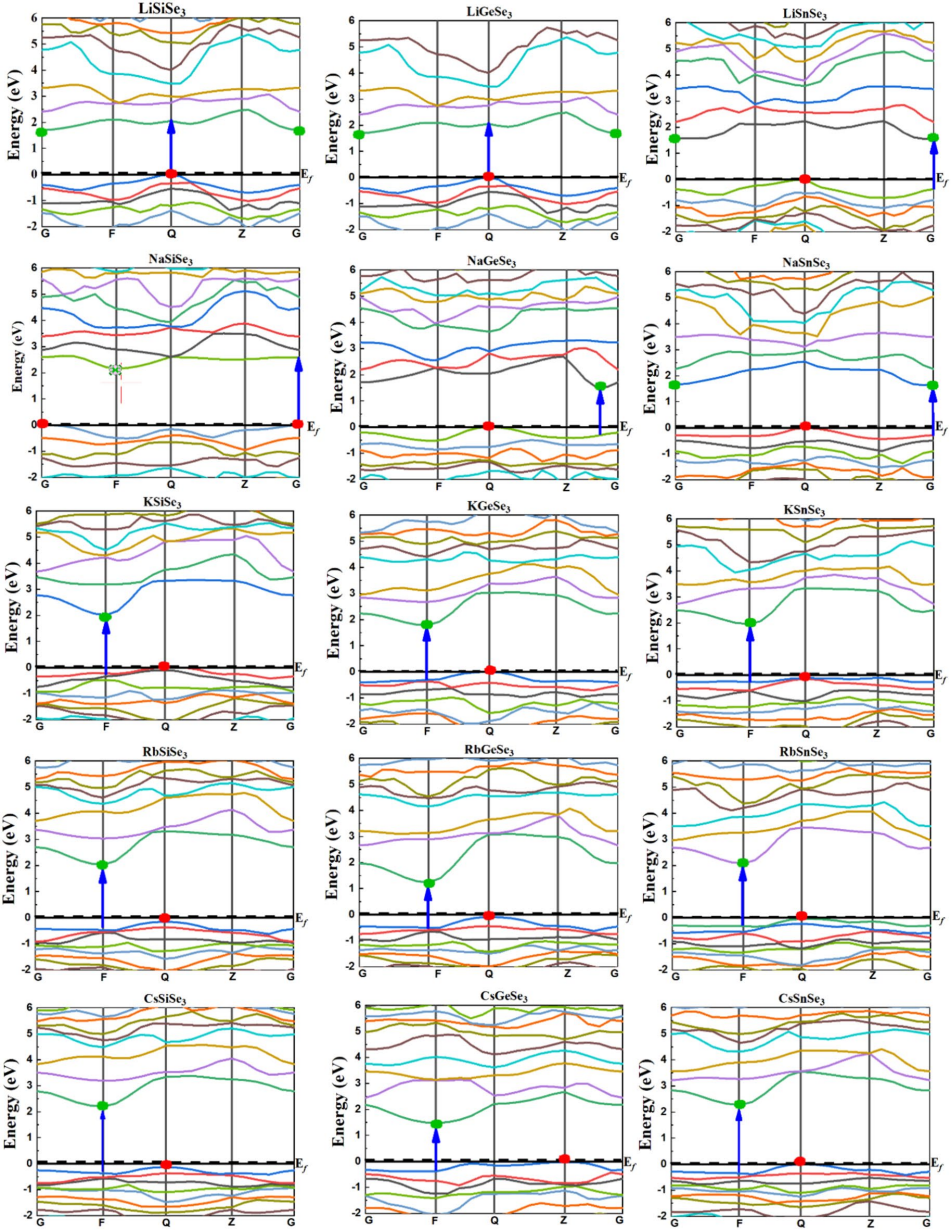
Band structures curves for the ABSe_3_ perovskites using the HSE06 method. The colored circles, red and green, on the highest valence and lowest conduction bands, represent the location of valance and conduction band edges, respectively. The blue arrow indicates the onset of the direct band.

Figure 3 displays the predicted electronic band structures (BS) for the investigated Si-based, Ge-based, and Sn-based chalcogenide perovskites. The calculated bandgap (*E*_g_) and the difference between indirect and direct gaps (Δ*E*_g_) for the investigated perovskites are summarized in Table 3. An indirect bandgap occurs when the maximum energy of the valence band (VB) and the minimum energy of the conduction band (CB) do not align at the same momentum (*k*-vector). This means that for an electron to transition from the VB to the CB, it must also interact with a phonon (a lattice vibration) to conserve momentum, making the process less efficient. This contrasts with direct bandgap materials, where such transitions can occur without the need for additional momentum exchange, allowing for more efficient photon absorption and emission. For solar cell applications, semiconductors with direct bandgaps are generally considered superior to those with indirect bandgaps. This is because direct bandgap materials exhibit significantly stronger optical absorption at the band edge due to the allowed momentum transitions between electrons and holes. Indirect semiconducting materials can also be efficient for solar cells, particularly when the Δ*E*_g_ falls within a specific range of approximately 0.2–0.5 eV^78^. A smaller Δ*E*_g_ in indirect bandgap semiconductors can potentially decrease electron–hole separation, avoiding fast recombination. According to recent research, the optimal band gap for solar cell applications should fall between 0.9 and 2.5 eV^31^. The BS reveal that all ABSe_3_ perovskites exhibit semiconducting behavior with indirect bandgaps. In general, the *E*_g_ increases with larger A-cations (Li to Cs) and follows the order Si > Sn > Ge for B-cations. The investigated perovskites exhibit indirect bandgaps within a range of 1.10–2.33 eV. This range indicates the tunability of the bandgap through compositional changes and is comparable to that of other perovskite structures and hybrid organic–inorganic compounds (Table 3). For instance, CH_3_NH_3_PbI_3_ is a widely studied perovskite that has an indirect band gap of approximately 1.6 eV^79,80^, which is within the range of the ABSe_3_ perovskites. This material is known for its strong absorption properties and is a leading candidate for solar cell applications. Recent studies have also explored lead-free perovskites, such as (C_6_H_5_NH_3_)BiI_4_, which have band gaps around 2.14 eV^81^. These materials are being investigated for their potential in photovoltaic applications while avoiding the toxicity associated with lead. Materials like YMnO_3_ and its derivatives have been reported to have band gaps around 1.35 eV^82^, which is close to the lower end of the ABSe_3_ perovskites’ band gap range. These materials also show promise for solar cell applications due to their suitable band gap energies. Furthermore, the ABSe_3_ possess a Δ*E*_g_ range of 0.149–0.493 eV, making them promising candidates for high-efficiency solar cell applications.

**Table 3:** The *E*_g_ and Δ*E*_g_ values of the studied perovskites using the HSE06 method compared with similar published perovskites.

Perovskite	*E*_g_	Δ*E*_g_	*E*_g_ type	Ref	Perovskite	*E*_g_	Method	*E*_g_ type	Refs.
LiSiSe_3_	1.69	0.405	Indirect	This work	(CH_3_NH_3_)PbI_3_	1.6	Exp.	Indirect	^79,80^
NaSiSe_3_	2.15	0.493	Indirect	This work	(C_6_H_5_NH_3_)BiI_4_	2.14	Exp.	Direct	^81^
KSiSe_3_	2.01	0.228	Indirect	This work	YMnO_3_	1.35	Exp.	Direct	^82^
RbSiSe_3_	2.18	0.317	Indirect	This work	CaSnS_3_	1.40	HSE06	Direct	^34^
CsSiSe_3_	2.33	0.265	Indirect	This work	BaHfS_3_	1.97	HSE06	Direct	^34^
LiGeS_3_	1.10	0.237	Indirect	This work	CaHfS_3_	2.23	HSE06	Direct	^34^
NaGeSe_3_	1.45	0.189	Indirect	This work	SrHfS3	2.32	HSE06	Direct	^34^
KGeSe_3_	1.78	0.311	Indirect	This work	Ba_2_CdS_3_	2.56	HSE06	Direct	^83^
RbGeSe_3_	1.32	0.418	Indirect	This work	Ba_2_CdSe_3_	2.16	HSE06	Direct	^83^
CsGeSe_3_	2.21	0.331	Indirect	This work	Ba_2_CdTe_3_	3.16	HSE06	Direct	^83^
LiSnS_3_	1.55	0.371	Indirect	This work	Ba_6_CS_4_	1.554	HSE06	Direct	^84^
NaSnSe_3_	1.63	0.288	Indirect	This work	Ba_6_CSe_4_	1.311	HSE06	Direct	^84^
KSnSe_3_	2.06	0.149	Indirect	This work	Ba_6_CTe_4_	1.517	HSE06	Direct	^84^
RbSnSe_3_	2.11	0.278	Indirect	This work	SrZrS_3_	2.009	TB-mBJ	Direct	^41^
CsSnSe_3_	2.27	0.352	Indirect	This work	SrZrSe_3_	1.096	TB-mBJ	Direct	^41^
					BaZrS3	1.770	TB-mBJ	Direct	^42^
					BaZrSe3	1.250	TB-mBJ	Direct	^42^

#### Density of states

**Fig. 4:**
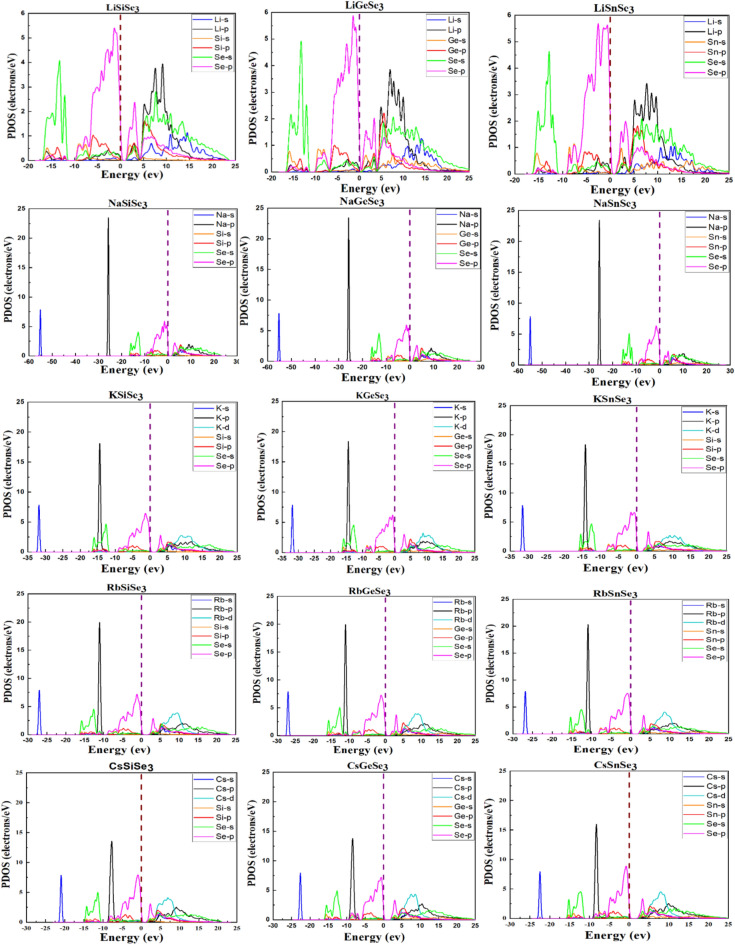
The calculated PDOS of ABSe_3_ perovskites using the HSE06, the maroon vertical dashed line represents the Fermi level (*E*_*F*_), the reference point with zero energy.

The density of states (DOS) signifies the number of electronic states possible at each energy level for electrons in a material. The DOS provides a general overview of the electrical structure without specifying which atomic orbitals contribute to various states. In contrast, the partial density of states (PDOS) divides the overall DOS into contributions from individual atomic orbitals or atoms. This allows researchers to investigate how individual atomic qualities affect the material’s electrical properties. The PDOS is derived by projecting the overall density of states onto specific atomic orbitals, such as *s*, *p*, or *d* orbitals, for each atom in the system, as shown in Fig. 4. Analysis of the PDOS of the VB of Li-based and K-based chalcogenides indicates that the states near the Fermi level (0 eV) down to − 10 eV are predominantly composed of Se-*p* states, with a moderate contribution from B-*p* states. A smaller contribution is observed from A-*p* and Se-*s* states, while A-*s* and B-*s* states contribute minimally. For K-based, Rb-based, and Cs-based chalcogenides, the contributions remain predominantly from the transition Se-*p* electrons, with a small contribution from B-*p* electrons and a minor contribution of Se-*s* electrons. The A-cation *s* states reveal the consistent presence of sharp peaks at around − 55 eV for all compounds in Na-based chalcogenides, − 32 eV for all compounds in K-based chalcogenides, − 27 eV for all compounds in Rb-based chalcogenides, − 21 eV for all compounds in Cs-based chalcogenides. The A-cation p states exhibit sharp peaks around − 25 eV for all compounds in Na-based chalcogenides, − 15 eV for all compounds in K-based chalcogenides, − 12 eV for all compounds in Rb-based chalcogenides, − 8 eV for all compounds in Cs-based chalcogenides. The states at the bottom of the CB are mainly due to the Se-*p* states. Analysis of the K-based, Rb-based, and Cs-based chalcogenides reveals that the *d* states of the A-cation exhibit broad peaks within the CB, ranging approximately from 3 to 18 eV.

### Optical properties

**Fig. 5:**
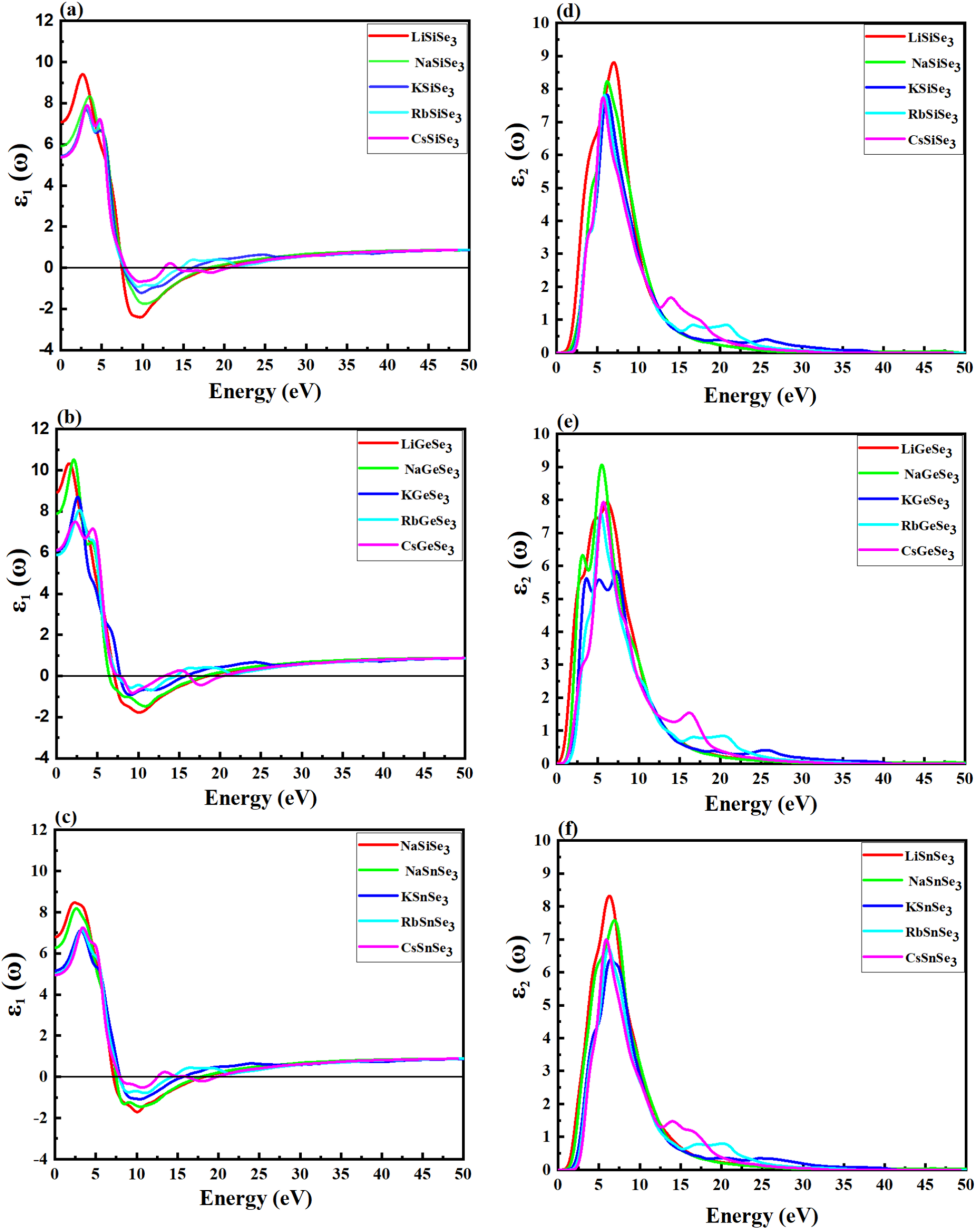
The calculated real dielectric, ε₁(ω), of (**a**) Si-based, (**b**) Ge-based, and (**c**) Sn-based chalcogenide perovskites using the HSE06; the imaginary dielectric, ε_2_(ω), of (**d**) Si-based, (**e**) Ge-based, and (**f**) Sn-based perovskites.

The optical properties of perovskites play a critical role in understanding their internal structures. The dielectric function, ε(ω), characterizes the interaction of an electromagnetic wave with a material as a function of frequency (ω). It describes how the material polarizes in response to the electric field of the wave. Furthermore, the dielectric function can also provide insights into the interactions between electrons and phonons within the material. The real part of the complex dielectric function, ε₁(ω), reflects the polarization induced in the material by the propagating light wave. While, the imaginary part, ε₂(ω), quantifies the energy absorbed by the material due to the interaction with the electromagnetic wave. Determination of the ε_1_(ω) and ε_2_(ω) allows us to calculate other optical properties like absorption, refractive index, dielectric function, conductivity, reflectivity, and energy loss. The ε_1_(ω) and ε_2_(ω) parts of dielectric constants are shown in Fig. 5. The ε_1_(ω) of LiSiSe_3_, NaSiSe_3_, KSiSe_3_, RbSiSe_3_ and CsSiSe_3_ at a photon energy of 0.01 are 7.08, 5.91, 5.43, 5.39, and 5.35, respectively (Fig. 5a). The ε_1_ (ω) at a photon energy of 0.01 for LiGeSe_3_, NaGeSe_3_, KGeS_3_, RbGeSe_3_ and CsGeSe_3_ are 8.930, 7.895, 6.085, 5.885, and 5.130, respectively (Fig. 5b). The ε_1_(ω) values at photon energy of 0.01 for LiSnSe_3_, NaSnSe_3_, KSnSe_3_, RbSnSe_3_ and CsSnSe_3_ are 6.789, 6.278, 5.160, 5.009 and 4.946, respectively (Fig. 5c). From the ε_1_(ω) values of Si-based, Ge-based, and Sn-based chalcogenides, we observe that the maximum ε_1_(ω) value decreases upon moving cation A from Li to Cs, which could be related to the increasing atomic size and decreasing electronegativity of the alkali metals. Comparing the three groups (Si, Ge, Sn) indicates how replacing Si with Ge or Sn alters the dielectric response. Ge-containing materials generally have higher ε₁(ω) values than their Si and Sn counterparts, indicating a stronger dielectric response, which might be crucial in applications where Ge is preferred for its superior electronic properties. Among the Si-based and Sn-based perovskites, the materials containing Li and Na exhibit higher values of the ε₁(ω). Materials with high ε₁(ω) at low energies are often more suitable for applications in optoelectronic devices, where a high dielectric constant is desirable for efficient charge separation and transport. LiGeSe_3_ and NaGeSe_3_ exhibit the highest ε_1_(ω) values at lower energies ~ (0.01 to 5.0) eV. Therefore, LiGeSe_3_ and NaGeSe_3_ exhibit competitive ε_1_(ω) values compared to traditional (~ 11.7 for silicon^85^, 9.5 for CdTe^86^) and emerging materials (~ 6.5 for CH_3_NH_3_PbI_3_^87^), suggesting their potential for effective use in solar cells, albeit with considerations for stability, cost, and scalability. For most Si-based, Ge-based, and Sn-based chalcogenides, ε_1_(ω) remains positive up to approximately 7.48–7.91 eV, 6.43–7.81 eV, and 7.15–7.95 eV, respectively. The ability to maintain positive ε₁(ω) at high energies means these materials are less likely to exhibit significant optical absorption losses in the visible and near-UV regions, which can enhance their performance in optoelectronic devices. The ε_2_(ω) starts increasing from zero at around 1.8 eV, which is an absorption edge and reaches its maximum value around 7.8 eV, which reveals that the absorption is maximum in the ultra-violet region.

**Fig. 6:**
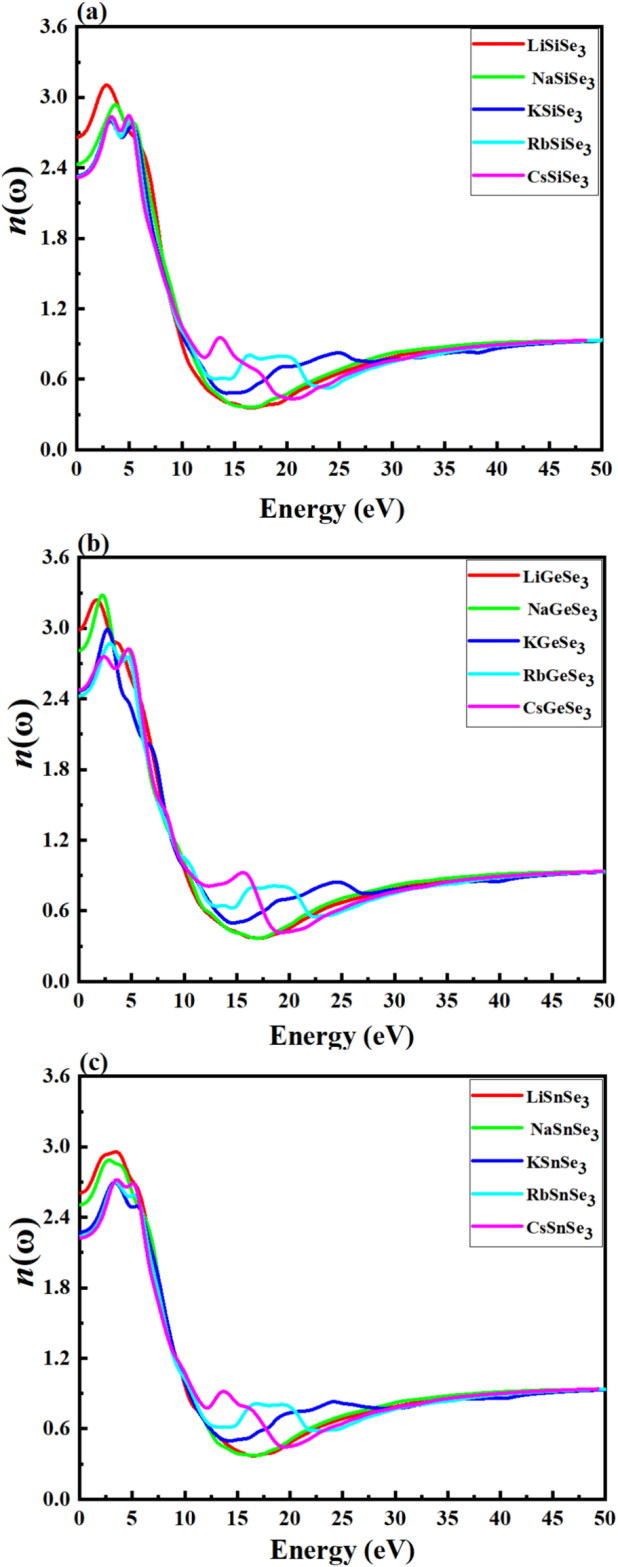
Calculated refractive index of (**a**) Si-based, (**b**) Ge-based, and (**c**) Sn-based chalcogenide perovskites using the HSE06 method.

**Fig. 7:**
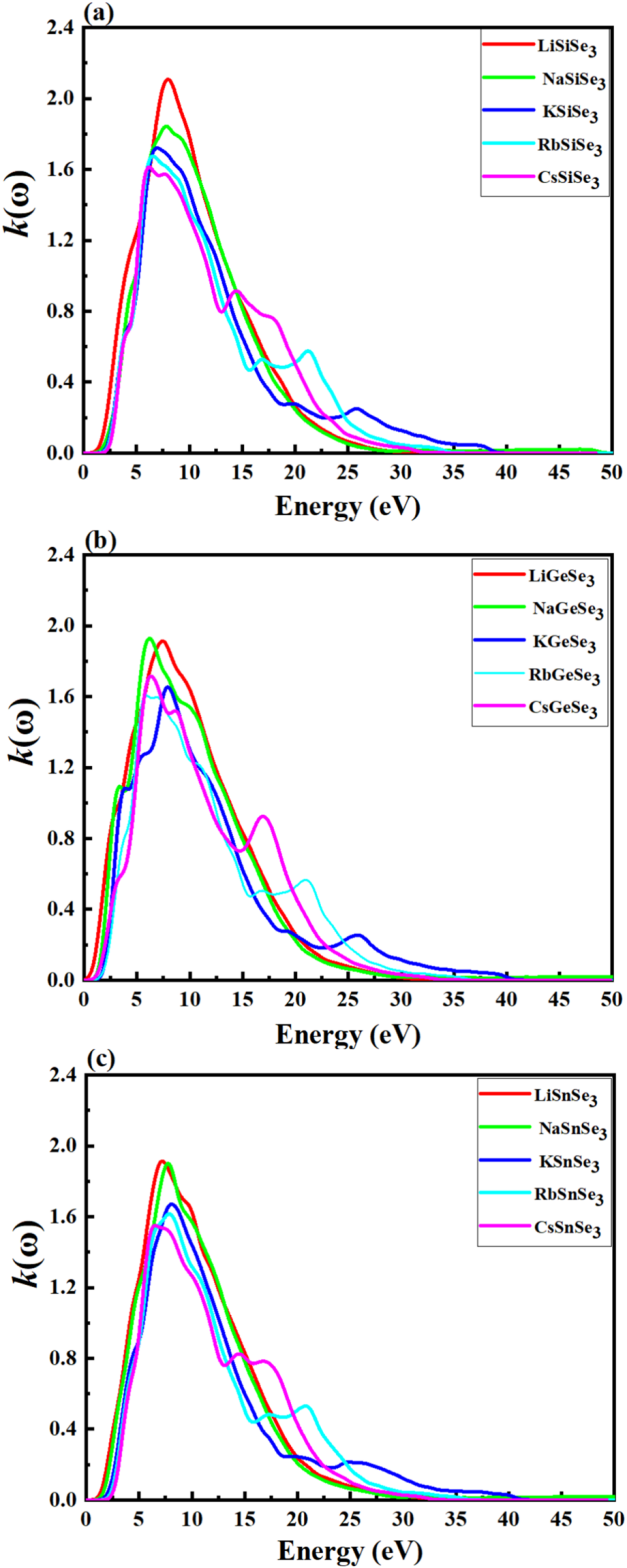
Calculated extinction coefficient of (**a**) Si-based, (**b**) Ge-based, and (**c**) Sn-based chalcogenide perovskites using the HSE06.

The refractive index *n*(ω) indicates the amount of light refracted or bent, when entering a material. Figure 6a–c represents the refractive index (real part) for (a) Si-based (b), Ge-based, and (c) Sn-based chalcogenides. Figure 6a depicts that LiSiSe_3_, NaSiSe_3_, KSiSe_3_, RbSiSe_3_ and CsSiSe_3_ have *n*(ω) of ∼ 2.661, 2.432, 2.330, 2.323, and 2.318 at photon energy of 0.01, respectively. From Fig. 6b, the *n*(ω) for LiGeSe_3_, NaGeSe_3_, KGeS_3_, RbGeSe_3_ and CsGeSe_3_ are 2.989, 2.809, 2.466, 2.425 and 2.475, respectively. The *n*(ω) values for LiSnSe_3_, NaSnSe_3_, KSnSe_3_, RbSnSe_3_, and CsSnSe_3_ are 2.605, 2.505, 2.271, 2.238 and 2.224, respectively (Fig. 6c). For each group (Si, Ge, Sn), the refractive index generally decreases as the cation size increases from Li to Cs. This trend suggests that larger cations lead to a lower refractive index, likely due to changes in lattice parameters and bonding characteristics. The Ge-based chalcogenides exhibit the highest refractive indices compared to Si and Sn-based materials. A higher refractive index can improve light trapping within the solar cell by increasing the likelihood of light being reflected and scattered within the active layer. This can enhance the absorption of light, especially in thin-film solar cells where the thickness of the absorbing material is limited. Given that LiGeSe_3_ and NaGeSe_3_ have the highest refractive indices among the materials listed, with values of 2.989 and 2.809 respectively. They are comparable to Si (~ 3.5)^88^ or slightly higher than CdTe (~ 2.6)^89^ and perovskites (CH_3_NH_3_PbI_3_, ~ 2.5–3.0)^90^. This suggests they are competitive with these well-known materials in terms of light interaction, which is significant for solar cell and optoelectronic applications. Figure 7a–c represents the extinction coefficient, *k*(ω), the imaginary part of the complex refractive index, for (a) Si-based, (b) Ge-based, and (c) Sn-based chalcogenides. It represents the attenuation or absorption of light as it propagates through a material. The local maxima peak of the *k*(ω) for our study perovskites centered around 8.0 eV. It is noted that Si-based chalcogenides exhibit the highest *k(*ω) compared with Ge-based and Si-based ones. Generally, the Li and Na-based chalcogenides tend to display higher values of the *n*(ω) and *k*(ω) compared to other materials in the same category.

**Fig. 8:**
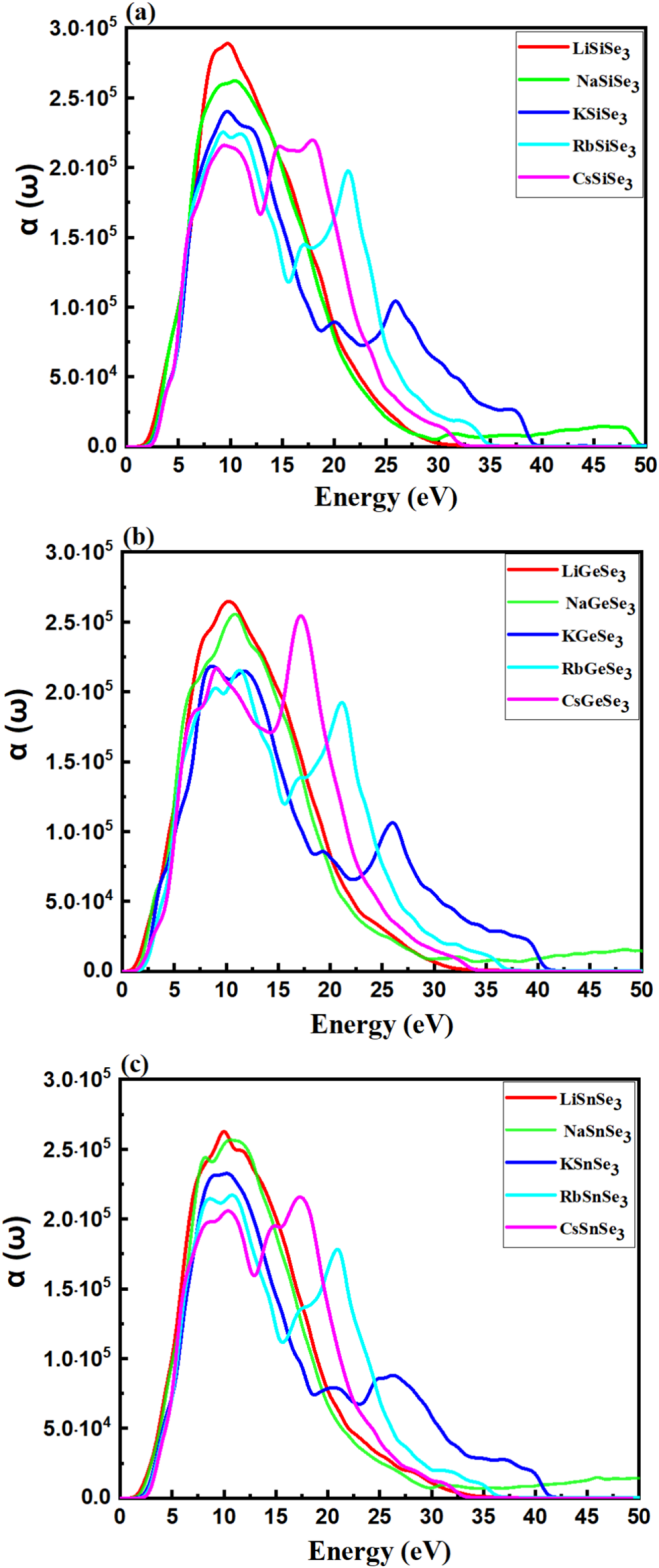
Calculated absorption coefficient of (**a**) Si-based, (**b**) Ge-based and (**c**) Sn-based perovskites.

Figure 8 shows the absorption coefficient $\alpha \left(\varepsilon \right)$ of chalcogenide perovskites. Within the 2–15 eV energy region, the absorption coefficient progressively decreases as the A-cation changes from Li to Cs. In the higher energy region (15 eV to 45 eV), the absorption coefficient slightly increases as the A-cation moves from K to Cs. Across the 2–45 eV energy range, the absorption coefficient generally decreases as the B-cation changes from Si to Sn. Notably, all the studied chalcogenide perovskites exhibit strong absorption in the UV region.

**Fig. 9:**
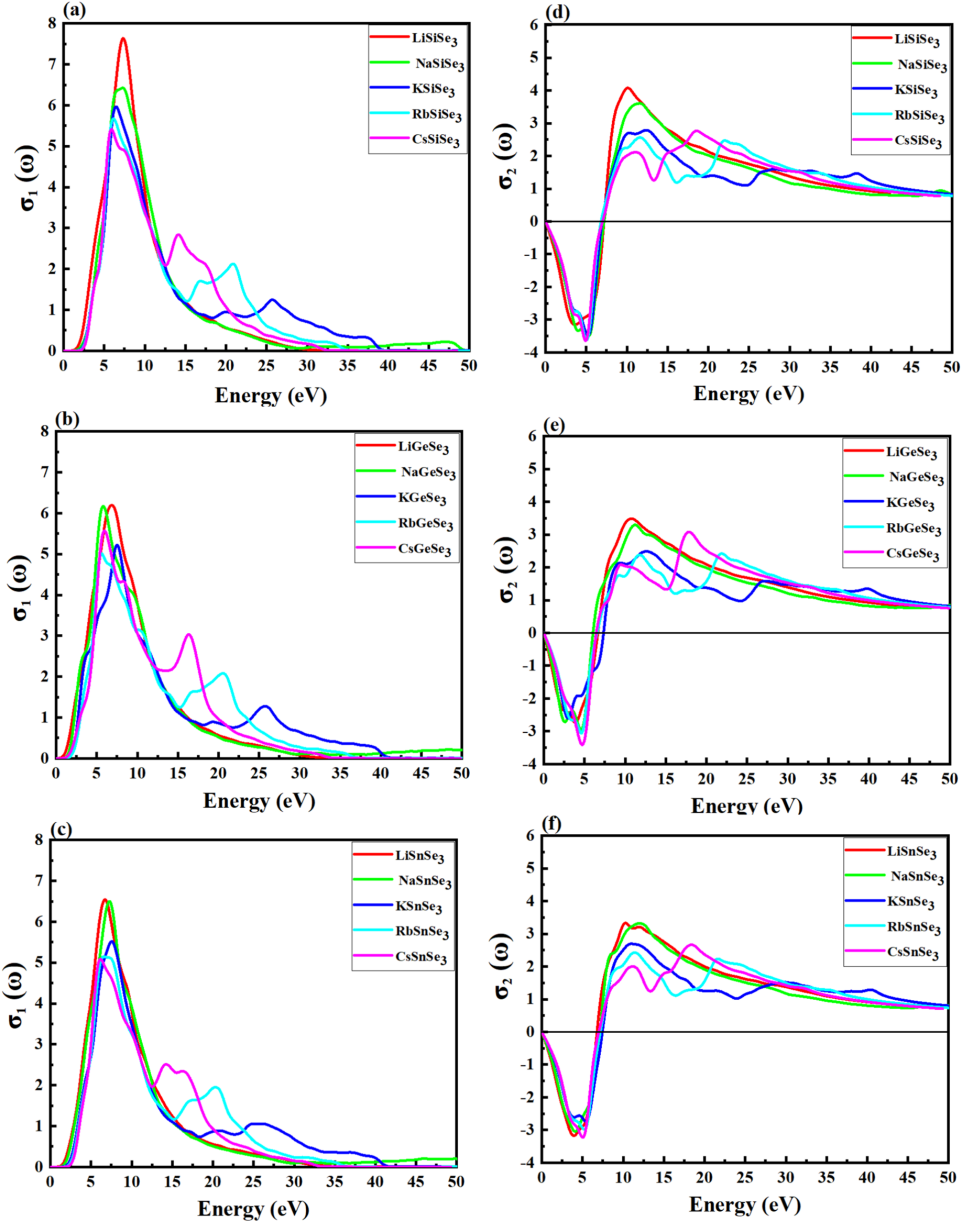
The calculated real part of optical conductivity for (**a**) Si-based, (**b**) Ge-based, and (**c**) Sn-based perovskites using the HSE09 method; the imaginary part of optical conductivity, (**d**) Si-based, (**e**) Ge-based, and (**f**) Sn-based perovskites.

The complex conductivity, σ(ω), describes the conduction of electrons due to an applied electromagnetic field. It is a complex function consisting of both real, σ_1_(ω), and imaginary component, σ_2_(ω). The σ_1_(ω) indicates how effectively a material can conduct electricity when subjected to electromagnetic radiation. Figure 9a–c illustrates the computed σ_1_(ω) spectra for studied perovskites at incoming photon energy ranging from 0 to 50 eV. All ABSe_3_ materials have a maximum σ_1_(ω) within the range of ~ 1–15 eV of photon energy, which is promising for their application in UV optical systems. This concurrence suggests that light absorption leads to the generation of free carriers, which in turn contribute to electrical conductivity. In this energy range, the σ_1_(ω) primarily corresponds to transitions involving Se-*p* electron with a moderate contribution of B-*p*, small of A-*p*, Se-*s*, and a minor contribution of A-*s* and B-*s* electrons for Li-based and Na-based chalcogenides. For K-based, Rb-based, and Cs-based chalcogenides, the contributions to the σ_1_(ω) remain predominantly from the transition Se-*p* electrons, with a small contribution from B-*p* electrons and a minor contribution of Se-*s* electrons. High σ_1_(ω) peaks were observed at ~ 16, 20, and 25 eV mainly corresponding to transitions concerning Se-p electron Ce-p, Rb-p, and K-p, respectively. Consistent with the absorption trends, the magnitude of the σ_1_(ω) spectra is higher for Si-based chalcogenide perovskites compared to Ge-based and Sn-based perovskites. The σ_2_(ω) represents the reactive component of the conductivity, which to the storage of energy in the material. The σ_2_(ω) curves extend from 0 eV incident photon energy up to 50 eV. At around 0 eV to 7 eV input photon energy, ABSe_3_ reach negative maximum values of about (− 3.14, − 3.489, − 3.480, − 3.619 and − 3.651) 1/*fs* for LiSiSe_3_, NaSiSe_3_, KSiSe_3_, RbSiSe_3_ and CsSiSe_3_ respectively (Fig. 9d). Negative maximum σ_2_(ω) values for LiGeSe_3_, NaGeSe_3_, KGeSe_3_, RbGeSe_3_, and CsGeSe_3_ were obtained at around − 2.733, − 2.947, − 3.00, − 3.074, and − 3.407 1/*fs*, respectively (Fig. 9e). Figure 9f reveals that for LiSnSe_3_, NaSnSe_3_, KSnSe_3_, RbSnSe_3_ and CsSnSe_3_ negative maximum σ_2_(ω) values at around − 3.170, − 3.045, − 2.741, − 2.996 and − 3.249 respectively. The negative maximum values of σ_2_(ω) in chalcogenide perovskites significantly impact their overall conductivity by reducing it and indicating a capacity for energy storage. The larger negative values in chalcogenide perovskites indicate that these materials may be more effective for applications requiring efficient energy storage and conversion, such as in solar cells. At around 7 eV to 15 eV input photon energy, Si-based, Ge-based, and Sn-based perovskites maximum positive of σ_2_ (ω) about range (2–4), (2.1–3.5) and (1.85–3.2) 1/*fs*. The shift to positive σ_2_(ω) values implies that charge carriers (electrons) are becoming more mobile and contributing to the conduction process.

**Fig. 10:**
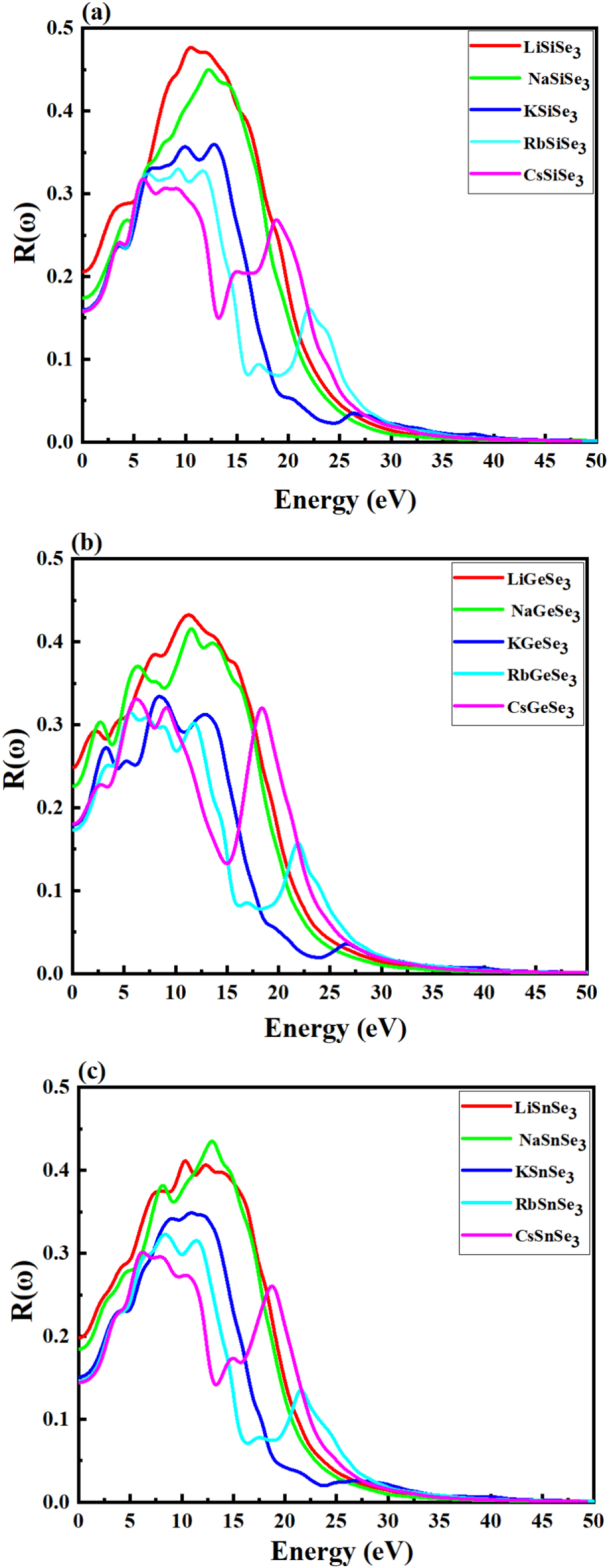
Reflectivity spectra of (**a**) Si-based, (**b**) Ge-based, and (**c**) Sn-based perovskites using the HSE06 method.

The reflectivity,* R*(ω), mainly measures the ability of a material to reflect incident light. Figure 10a–c depicts the *R*(ω) values for ABSe_3_ at a photon energy of 0.01 eV. The *R* (ω) values for LiSiSe_3_, NaSiSe_3_, KSiSe_3_, RbSiSe_3_, and CsSiSe_3_ are ~ 0.205, 0.174, 0.159, 0.158, and 0.157, respectively (Fig. 10a). Similarly, for LiGeSe_3_, NaGeSe_3_, KGeSe_3_, RbGeSe_3_, and CsGeSe_3_, the R(ω) values are 0.248, 0.225, 0.179, 0.173, and 0.180, respectively (Fig. 10b). Finally, Fig. 10c shows that the R(ω) values for LiSnSe3, NaSnSe_3_, KSnSe_3_, RbSnSe_3_, and CsSnSe_3_ are 0.198, 0.184, 0.151, 0.146, and 0.144, respectively. For efficient solar cells, lower reflectivity is desirable because it means that more incident light is absorbed rather than reflected. Across all groups (Si-based, Ge-based, and Sn-based), the reflectivity *R*(ω) generally tends to decrease as we move from Li-based to Cs-based chalcogenides. This trend suggests that as the atomic number of the A cation increases, the material becomes slightly more transparent or less reflective at the given photon energy (0.01 eV). Among the different groups, Ge-based chalcogenides generally exhibit higher reflectivity values than Si-based and Sn-based chalcogenides, at 0.01 eV. This implies that Ge-based chalcogenides may reflect more light, which could reduce their effectiveness in absorbing photons and thus converting them into electrical energy in solar cell applications. Materials like CsSnSe_3_, which have the lowest reflectivity (~ 0.144), are therefore more favorable for solar cell applications as they would absorb lighter and potentially generate more electricity. While the Ge-based ABSe_3_ chalcogenides show slightly higher reflectivity, they still fall within a reasonable range that could be optimized through material engineering (e.g., surface texturing, anti-reflective coatings) for better solar cell performance. The reflectivity of the ABSe_3_ materials, particularly the Sn-based compounds, is comparable to or lower than that of some well-known materials used in thin-film solar cells, such as Cu₂ZnSnS₄ (20–25% in the visible spectrum)^91^, SrTiO_3_ (R around 20–30% in the visible spectrum)^92^, and CH_3_NH_3_PbI_3_ (5–15% in the visible spectrum)^93^. This low reflectivity makes ABSe_3_ materials potential candidates for solar applications. The reflectivity is more significant in the range of 1.2 eV to 35 eV and becomes negligible at above 45 eV.

**Fig. 11:**
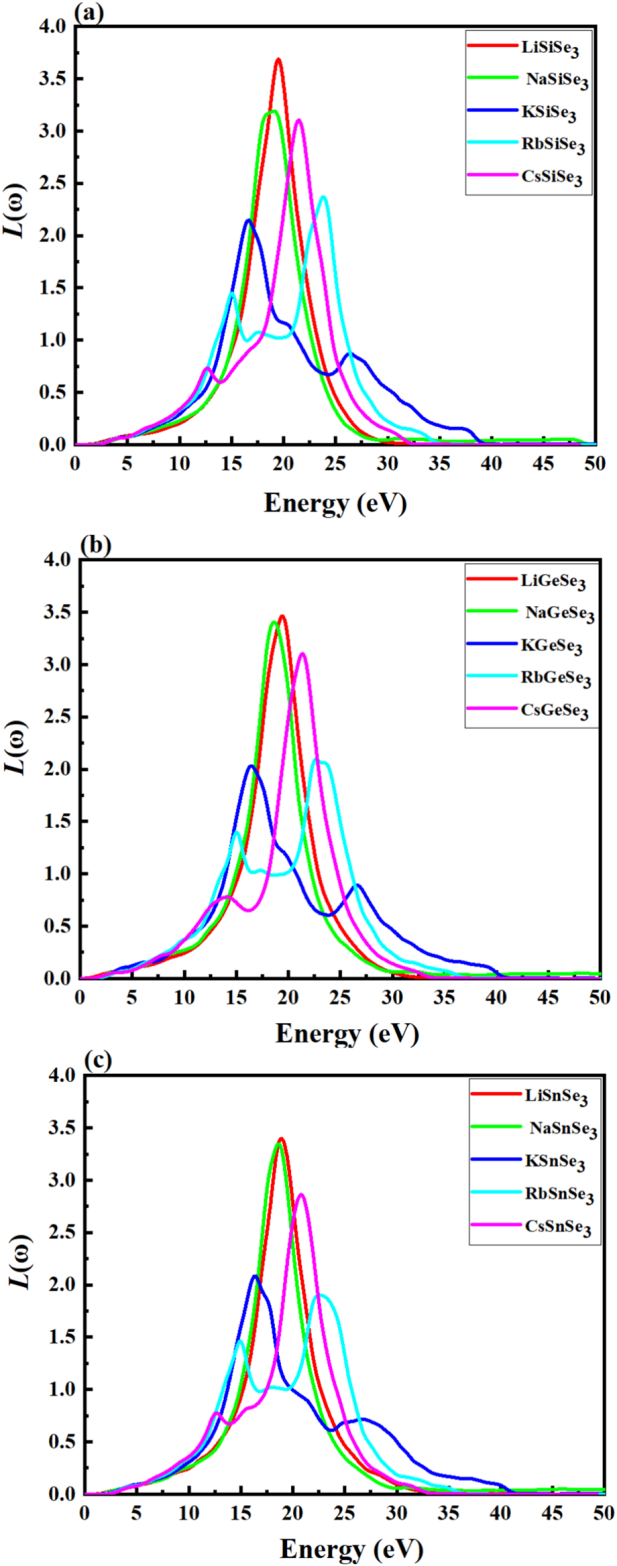
The optical loss function of (**a**) Si-based, (**b**) Ge-based, and (**c**) Sn-based perovskites using the HSE06 method.

The electron loss energy, L(ω), partly describes the energy loss due to fast-moving electrons through the lattice. Figure 11 reveals that for the perovskite materials, L(ω) is zero for photon energies between 0 and 4.48 eV. In Fig. 11a, a sharp peak is found at 19.5, 18.9, 16.7, 23.9 and 21.5 eV for LiSiSe_3_, NaSiSe_3_, KSiSe_3_, RbSiSe_3_ and CsSiSe_3_, respectively. Figure 11b also exhibits sharp peaks in the L(ω) spectra for Ge-based perovskites. The peak positions show variations across the A-cation (Li, Na, K, Rb, Cs), with values of 19.4, 18.6, 16.8, 22.8, and 21.4 eV, respectively. Similarly, the specific peak positions for LiSnS_3_, NaSnS_3_, KSnS_3_, RbSnS_3_, and CsSnS_3_ are 18.9, 18.7 eV, 16.4 eV, 22.8 eV, and 20. 8 eV, respectively (Fig. 11c). The LiSiS_3_, LiGeS_3_ and LiSnS_3_ perovskites show highest L(ω) values, while KSiS_3_, KGeS_3_, and RbSnS_3_ show lowest peaks compared to the others perovskites.

## Conclusions

This study employs density functional theory calculations to comprehensively investigate the structural, electronic, and optical properties of triclinic chalcogenide perovskites, ABSe_3_ (A = Li, Na, K, Rb, Cs; B = Si, Ge, Sn). Our findings reveal several important insights: Firstly, the tolerance factor and negative formation energy values suggest that these compounds exhibit both thermodynamic and structural stability, indicating their potential for practical applications. Secondly, the absence of imaginary phonon modes in the phonon dispersion curves confirms the dynamical stability of ABSe_3_ perovskites, eliminating concerns about structural instability in practical applications. Thirdly, examination of the band structures indicates all studied perovskites exhibit favorable indirect band gaps (1.10–2.33 eV) with a small difference from direct gaps (0.149–0.493 eV), providing potential applications, particularly in photovoltaics. A larger A-cation (Li to Cs) generally leads to a wider band gap, while for B-cations, *E*_g_ follows the order Si > Sn > Ge. Finally, the predicted optical properties of the proposed chalcogenide perovskites are comparable to some of the traditional and emerging materials. LiGeSe_3_ and NaGeSe_3_ exhibit the highest ε_1_(ω) values at lower energies ~ (0.01 to 5.0) eV. Therefore, LiGeSe_3_ and NaGeSe_3_ exhibit competitive ε1(ω) values compared to traditional (Si, CdTe) and emerging materials (e.g., CH_3_NH_3_PbI_3_). They also have the highest *n*(ω) among the ABSe_3_ materials listed, with close to Si and slightly higher than CdTe and CH_3_NH_3_PbI_3_. The ABSe_3_ materials exhibit a high absorption coefficient within the energy range of ~ 2–15 eV, and their reflectivity, particularly the Sn-based compounds (e.g., CsSnSe_3_), are comparable to or lower than that of some well-known materials used in thin-film solar cells, such as Cu₂ZnSnS₄, SrTiO_3_, and CH_3_NH_3_PbI_3_. The properties suggest ABSe_3_ could be promising candidate materials for solar cell applications. Future research should focus on experimentally validating the predicted properties of ABSe_3_ perovskites through synthesis, characterization, and optical/electronic measurements. Exploring other compositions, such as doping and mixed systems, could further optimize their properties. Additionally, simulating and developing prototypes for photovoltaic devices will help assess the real-world efficiency of these materials. Finally, studying their environmental stability and toxicity is crucial for ensuring safe and practical applications.

## Data Availability

All data generated or analysed during this study are included in this published article.
